# Synergistic Tumor Inhibition via Energy Elimination by Repurposing Penfluridol and 2-Deoxy-D-Glucose in Lung Cancer

**DOI:** 10.3390/cancers14112750

**Published:** 2022-06-01

**Authors:** Tsung-Ching Lai, Yueh-Lun Lee, Wei-Jiunn Lee, Wen-Yueh Hung, Guo-Zhou Cheng, Ji-Qing Chen, Michael Hsiao, Ming-Hsien Chien, Jer-Hwa Chang

**Affiliations:** 1 Division of Pulmonary Medicine, Department of Internal Medicine, Wan Fang Hospital, Taipei Medical University, Taipei 11696, Taiwan; 109053@w.tmu.edu.tw; 2 Pulmonary Research Center, Wan Fang Hospital, Taipei Medical University, Taipei 11696, Taiwan; 3 Department of Microbiology and Immunology, School of Medicine, College of Medicine, Taipei Medical University, Taipei 11031, Taiwan; yllee@tmu.edu.tw; 4 Graduate Institute of Clinical Medicine, College of Medicine, Taipei Medical University, Taipei 11031, Taiwan; lwj5905@gmail.com (W.-J.L.); hungwenyueh@yahoo.com.tw (W.-Y.H.); a29378488@gmail.com (G.-Z.C.); 5 Department of Medical Education and Research, Wan Fang Hospital, Taipei Medical University, Taipei 11696, Taiwan; 6 Department of Urology, School of Medicine, College of Medicine, Taipei Medical University, Taipei 11031, Taiwan; 7 Department of Cancer Biology, Geisel School of Medicine at Dartmouth, Lebanon, NH 03755, USA; ji-qing.chen.gr@dartmouth.edu; 8 Genomics Research Center, Academia Sinica, Taipei 11529, Taiwan; mhsiao@gate.sinica.edu.tw; 9 TMU Research Center of Cancer Translational Medicine, Taipei Medical University, Taipei 11031, Taiwan; 10 Traditional Herbal Medicine Research Center, Taipei Medical University Hospital, Taipei 110301, Taiwan; 11 School of Respiratory Therapy, College of Medicine, Taipei Medical University, Taipei 11031, Taiwan

**Keywords:** lung cancer, glycolysis, mitochondria, SITR1, PGC-1α

## Abstract

**Simple Summary:**

Drug repurposing has been effective for discovering novel treatments for cancer. The antipsychotic agent penfluridol was reported to suppress lung cancer growth via ATP energy deprivation. The aim of our study was to investigate how penfluridol influences energy metabolism in lung cancer cells. We observed that penfluridol inhibited mitochondrial oxidative phosphorylation (OXPHOS), but induced glycolysis to compensate for the loss of ATP caused by suppression of mitochondrial OXPHOS. We also confirmed that inhibition of glycolysis by 2-deoxy-D-glucose (2DG) significantly augmented the antitumor effects caused by penfluridol in vitro and in vivo. Our studies provide novel insights into repurposing penfluridol combined with 2-DG for lung cancer treatment.

**Abstract:**

Energy metabolism is the basis for cell growth, and cancer cells in particular, are more energy-dependent cells because of rapid cell proliferation. Previously, we found that penfluridol, an antipsychotic drug, has the ability to trigger cell growth inhibition of lung cancer cells via inducing ATP energy deprivation. The toxic effect of penfluridol is related to energy metabolism, but the underlying mechanisms remain unclear. Herein, we discovered that treatment of A549 and HCC827 lung cancer cells with penfluridol caused a decrease in the total amount of ATP, especially in A549 cells. An Agilent Seahorse ATP real-time rate assay revealed that ATP production rates from mitochondrial respiration and glycolysis were, respectively, decreased and increased after penfluridol treatment. Moreover, the amount and membrane integrity of mitochondria decreased, but glycolysis-related proteins increased after penfluridol treatment. Furthermore, we observed that suppression of glycolysis by reducing glucose supplementation or using 2-deoxy-D-glucose (2DG) synergistically enhanced the inhibitory effect of penfluridol on cancer cell growth and the total amount of mitochondria. A mechanistic study showed that the penfluridol-mediated energy reduction was due to inhibition of critical regulators of mitochondrial biogenesis, the sirtuin 1 (SIRT1)/peroxisome-proliferator-activated receptor co-activator-1α (PGC-1α) axis. Upregulation of the SIRT1/PGC-1α axis reversed the inhibitory effect of penfluridol on mitochondrial biogenesis and cell viability. Clinical lung cancer samples revealed a positive correlation between PGC-1α (PPARGC1A) and SIRT1 expression. In an orthotopic lung cancer mouse model, the anticancer activities of penfluridol, including growth and metastasis inhibition, were also enhanced by combined treatment with 2DG. Our study results strongly support that a combination of repurposing penfluridol and a glycolysis inhibitor would be a good strategy for enhancing the anticancer activities of penfluridol in lung cancer.

## 1. Introduction

Lung cancer is the leading lethal cancer in men and women worldwide [1]. In total, ~85% of lung cancer cases are non-small cell lung cancer (NSCLC). It causes about 2 million deaths every year, and only 19% of patients survive longer than 5 years. In the previous decade, although developments in targeted therapy and immunotherapy have made great progress in treating lung cancer, there are still many patients who relapse after these treatments or are not suitable for these treatments and only can rely on radiotherapy and chemotherapy [2]. However, lung cancers are typically resistant to chemotherapy due to molecular modifications, such as induction of antiapoptotic genes or metabolic alterations, and activation of several DNA repair mechanisms that drive the active efflux of drugs from the cell cytoplasm [3]. Due to the unsatisfactory results of these standard treatments for NSCLC, identifying new agents is crucial.

Cancer cells exhibit metabolic reprogramming to provide sufficient energy and biosynthesis for cell proliferation, invasion, and migration [4]. Adenosine triphosphate (ATP) energy is mainly generated by glycolysis and mitochondrial oxidative phosphorylation (OXPHOS) from glucose in cells. According to the classical model of cancer bioenergetics, tumor cells mainly use glycolysis as the major source of ATP, and this phenomenon is called the Warburg effect [5]. Based on metabolic differences between tumor and normal cells, targeting glycolysis by a non-metabolizable glucose analog, 2-deoxy-D-glucose (2DG), is regarded as a promising strategy for eliminating tumor cells [6]. Recently, a growing number of studies have provided evidence that not all cancer cells depend on glycolysis; moreover, OXPHOS is also essential for supplying ATP and biosynthetic intermediates (such as NADH and NAD+) to support tumorigenesis, especially in KRAS-mediated tumorigenesis [7,8]. Among numerous regulators of cancer metabolism, peroxisome-proliferator-activated receptor co-activator-1α (PGC-1α) is emerging as an essential controller of multiple metabolic pathways. Recently, PGC-1α was reported to primarily regulate mitochondrial respiration and biogenesis in cancer cells [9]. Notably, PGC-1α is controlled by several posttranslational modifications. For example, it is tightly regulated by sirtuin 1 (SIRT1) by the deacetylation of lysine residues to modulate the glucose homeostasis [10,11]. Recently, interactions between SIRT1 and PGC-1α have attracted much attention in the study of tumorigenesis, as an SIRT1/PGC-1α-dependent increase in mitochondrial biogenesis and OXPHOS enhanced the chemoresistance of colon cancer and metastasis of hepatocellular carcinoma (HCC) [12,13].

In recent years, several non-anticancer drugs have been repurposed for cancer treatment, such as metformin for colorectal cancer [14], and nitroxoline for pancreatic cancer [15], suggesting that drug repurposing is a promising strategy in translational medicine for cancer treatment. Recently, repurposing antipsychotic medications for cancer therapy stemmed from multiple clinical studies which demonstrated that patients taking antipsychotic medications had lower cancer incidences [16]. Penfluridol is a long-acting oral antipsychotic drug used for treating schizophrenia [17] and was recently reported to exhibit anticancer activities in several cancer types [18,19]. Multiple mechanisms are reported to be involved in the anticancer activities of penfluridol on various cancers, such as modulating immune responses [20], suppressing cancer stemness [21], and inducing dysregulation of cholesterol homeostasis [22]. Our previous study found that penfluridol induced non-apoptotic cell death with features of autophagosome accumulation in lung cancer cells and an orthotopic xenograft model [23]. In addition, we observed that autophagosome accumulation-mediated cell growth inhibition by penfluridol was mainly attributed to ATP energy deprivation, but the underlying mechanism remained unclear.

In the present study, we tried to dissect the effect of penfluridol on the metabolic interplay between OXPHOS and aerobic glycolysis in two NSCLC cell lines (A549 and HCC827), which, respectively, harbor the mutant RAS and mutant epidermal growth factor receptor (EGFR). We further explored the possibility of manipulating metabolic reprogramming to enhance the penfluridol-mediated anticancer effect in lung cancer.

## 2. Materials and Methods

### 2.1. Reagents, Chemical Inhibitors, and Antibodies

Penfluridol (P3371), 2DG (D8375), ATP (A2383), dimethyl sulfoxide (DMSO), and other chemicals used in this study were purchased from Sigma-Aldrich (St. Louis, MO, USA). A 40 mM stock solution of penfluridol was made in DMSO, and the final concentration of DMSO for all treatments was <0.5%. JC-1 and MitoTrackr probes were purchased from ThermoFisher Scientific (Waltham, MA, USA). Primary antibodies, including hexokinase (HK, #2024), phosphofructokinase, platelet (PFKP, #8164), pyruvate kinase M2 (PKM2, #4053), pyruvate dehydrogenase (PDH, #2784), glyceraldehide-3-phosphate dehydrogenase (GAPDH, #2118), and phosphorylated (p-) and unphosphorylated forms of adenosine monophosphate (AMP)-activated protein kinase (AMPK, #2535 and #5832) were obtained from Cell Signaling Technology (Danvers, MA, USA). SIRT1 (sc-74504), PGC-1α (sc-517380), and β-actin (sc-47778) antibodies were obtained from Santa Cruz Biotechnology (Santa Cruz, CA, USA).

### 2.2. Cell Lines and Cell Culture

Human NSCLC cell lines (A549 and HCC827) with a RAS or EGFR mutation were purchased from the American Type Culture Collection (ATCC; Manassas, VA, USA) and grown in RPMI 1640 medium containing 10% fetal bovine serum (FBS) (Life Technologies, Grand Island, NY, USA) and a 1% penicillin, streptomycin, and glutamine mixture. All cells were grown in a humidified incubator at 37 °C with 5% CO_2_ and routinely tested for mycoplasma contamination.

### 2.3. Cell Viability and Clonogenic Assays

To detect the cell proliferation rate, NSCLC cells (5 × 10^3^) were seeded in 96-well plates and incubated overnight. Cells were next treated with penfluridol alone or with 2DG for the indicated time point. At the end of the incubation period, the cell viability was determined by an MTS cell viability assay (Promega, Madison, WI, USA) according to the manufacturer’s instructions. Data were collected from three replicates. The combination index (CI) was calculated by CompuSyn software indicating the effects of drug combinations [24]. For clonogenic assays, 1000 NSCLC cells were plated in six-well plates and incubated for 24 h. Cells were then treated with indicated compounds such as penfluridol and ATP for 24 h, and then continuously incubated in new fresh medium containing ATP at 37 °C for 7–10 days. Colonies were then fixed with 4% paraformaldehyde and stained with crystal violet (0.5% *w/v*), and colonies were manually counted using ImageJ software (National Institutes of Health, Bethesda, MD, USA).

### 2.4. Intracellular ATP Detection Assay

Briefly, 5000 NSCLC cells were seeded into a 96-well plate for 24 h and treated with various concentrations of penfluridol for another 24 h. Cells were lysed, and then intracellular ATP was detected according to the manufacturer’s instructions in the manual of the luminescent ATP detection assay kit (Abcam, Cambridge, MA, USA).

### 2.5. ATP Production Rate of Glycolytic and Mitochondrial OXPHOS

The ATP production rate was detected with a Seahorse XF Real-Time ATP Rate Assay Kit (Seahorse Bioscience, Lexington, MA, USA). Real-time measurements were made using an XF-96 Extracellular Flux Analyzer (Seahorse Bioscience). Briefly, NSCLC cells were plated in XF-96 plates (Seahorse Bioscience) at a concentration of 4 × 10^4^ cells/well and cultured overnight. Cells were treated with penfluridol (1.25~5 μM) for 24 h. Each well was washed with a Dulbecco’s modified Eagle medium (DMEM) solution supplemented with 10 mM glucose, 2 mM L-glutamine, and 1 mM sodium pyruvate before the start of the experiment. Baseline measurements of the oxygen consumption rate (OCR, measured by a change in the oxygen concentration) and extracellular acidification rate (ECAR, measured by a change in the pH) were taken before sequential injection of treatments/inhibitors: oligomycin (an ATP synthase inhibitor) and antimycin A and rotenone (electron transport blockers). OCR and ECAR data were calculated to determine ATP production rates using the Seahorse XF Real-Time ATP Rate Assay Report Generator.

### 2.6. NAD^+^ Measurement

NAD^+^ levels were measured using a NAD^+^/NADH Quantification Colorimetric kit (Biovision Inc, Milpitas, CA, USA), following the manufacturer’s instructions. Briefly, 2 × 10^5^ NSCLC cells were treated with various concentrations of penfluridol for 24 h, and cells were collected in 400 μL of NAD^+^/NADH extraction buffer. Samples from each treatment group were separated into two sets, one of which was used to perform a thermal decomposition assay of NAD^+^ followed by an enzymatic cycling assay to determine the NADH content. The other set was used to measure the total NAD (NADH plus NAD^+^) content by performing a cycling assay without decomposition of NAD^+^. The absorbance of the final products was measured at 450 nm, and absolute NAD concentrations were further calculated. The NAD^+^/NADH ratio is presented as the percentage of (total NAD-NADH)/NADH.

### 2.7. Measurement of the Mitochondrial Mass

NSCLC cells were treated with penfluridol for 24 h in the absence and presence of 2DG. The mitochondrial mass of NSCLC cells was determined by selectively loading mitochondria with the red fluorescent dye, MitoTracker Red. Briefly, compound-treated cells were stained with 20 nM MitoTracker Red for 15 min at 37 °C and analyzed by a FACScan laser flow cytometric analysis system (Beckman Coulter, Hialeah, FL, USA).

### 2.8. Total Cell Protein Lysate Extraction and Western Blot Analysis

Vehicle- and penfluridol-treated cells were harvested and lysed with RIPA buffer (ThermoFisher Scientific, Rockford, IL, USA) containing protease inhibitors (Sigma-Aldrich, St. Louis, MO, USA). The protein content was determined with the Bio-Rad protein assay reagent using bovine serum albumin as a standard. Detailed processes of the Western blot analysis were previously described [25]. The primary antibodies used in Western blot analysis included HK, PFKP, PKM2, PDH, SIRT1, PGC-1α, p53, p-AMPK, AMPK, and GAPDH.

### 2.9. Mitochondrial Membrane Potential (MMP) Assessment

Breakdown of the MMP was assessed with a Zeiss Axiophot fluorescence microscope (Carl Zeiss, Thornwood, NY, USA) using 5,5,6,6′-tetrachloro-1,1′,3,3′-tetraethylbenzimi-dazoylcarbocyanine iodide (JC-1) dye, which allows detection of changes in the MMP. A549 cells were treated with penfluridol for 24 h and further incubated for 15 min in a freshly prepared JC-1 solution (5 µg/mL). Spare dye was removed by washing with phosphate-buffered saline (PBS), and cell-associated fluorescence was measured with fluorescent microscopy. In the case of mitochondrial depolarization, JC-1 is present in the cytoplasm as monomers and manifests as green signals.

### 2.10. Bioinformatics Analysis

cBioportal platform (https://www.cbioportal.org/, accessed on 2 April 2022) was used to analyze the correlations between *PPARGC1A* and indicated genes from the lung adenocarcinoma (TCGA, PanCancer Atlas) dataset including 566 cases. mRNA expression Z-scores relative to normal samples (logRNA Seq V2 RSEM) were chosen for the mRNA expression of genomic profiles.

### 2.11. Gene Expression Analysis by a Real-Time Quantitative Polymerase Chain Reaction (RT-qPCR) Analysis

Cellular messenger (m)RNA was isolated with the TRIzol reagent (Invitrogen Life Technologies, Carlsbad, CA, USA). Complementary (c)DNA synthesis was performed using an iScript™ cDNA Synthesis kit (Bio-Rad Laboratories, Irvine, CA, USA) followed by gene amplification using OmicsGreen 5X qPCR Master Mix with a StepOnePlus™ Real-Time PCR System (Applied Biosystems, Carlsbad, CA, USA). Gene expressions were normalized using ΔCt relative to GAPDH. Expression changes were expressed using the 2^−ΔΔCt^ method [26]. Primer sequences used in this study were as follows: PPARGC1A forward, GTGAAGACCAGCCTCTTTGC, and reverse, AATCCGTCTTCATCCACAGG; mitochondrially encoded cytochrome C oxidase 1 (MTCO1) forward, ACGTTGTAGCCCACTTCCAC, and reverse, CATCGGGGTAGTCCGAGTAA; MTCO2 forward, TTCATGATCACGCCCTCATA, and reverse, TAAAGGATGCGTAGGGATGG; mitochondrially encoded NADH:ubiquinone oxidoreductase core subunit 6 (MTND6) forward, TGATTGTTAGCGGTGTGGTC, and reverse, CCACAGCACCAATCCTACCT; and GAPDH forward, AGTCAGCCGCATCTTCTTTTG, and reverse, CCACTTGATTTTGGAGGGATCT.

### 2.12. Lung Orthotopic Xenograft Tumor Model

Six-week-old nonobese diabetic (NOD)-SCID mice were used in assays for lung tumor growth and metastasis in an orthotopic graft model. All animal experiments were performed under a protocol approved by the Institutional Animal Care and Use Committee of Wan Fang Hospital, Taipei Medical University (no. WAN-LAC-109-002). In brief, 5 × 10^5^ luciferase-tagged A549 cells (A549-Luc) were mixed with Matrigel and directly injected into the left lung parenchyma of NOD-SCID mice. The cancer cell injection day represented day 0. After 7 days, mice were randomized into experimental and control groups according to bioluminescence imaging (BLI) results from the Xenogen IVIS spectrum system (Xenogen, Alameda, CA, USA), and orally given vehicle, 5 mg/kg penfluridol or combined with the intraperitoneal injection of 100 mg/kg 2DG 5 days per week. After 4 weeks, mice were sacrificed, and ex vivo images of tumor-bearing tissues including the lungs, kidneys, liver, and pancreas excised from the mice were carried out in an IVIS Spectrum system. Tumor specimens from the lungs were harvested, photographed, and subjected to hematoxylin and eosin (H&E) staining for further histopathological analyses.

### 2.13. Immunohistochemical (IHC) Staining

The processes of IHC staining were previously described [23]. Briefly, paraffin-embedded tumor tissues were deparaffinized with xylene and incubated with 0.3% H_2_O_2_ to block endogenous peroxidase activity. Slides were washed with PBS and incubated with anti-PGC-1α (1:20), anti-SIRT1 (1:50), or anti-Ki67 (1:100) antibodies for 2 h at room temperature. After washing in PBS, slides were developed with a VECTASTAIN ABC peroxidase kit (Vector Laboratories, Burlingame, CA, USA) and a DAB peroxidase substrate kit (Vector Laboratories) according to the manufacturer’s instructions. All specimens were deparaffinized and stained with hematoxylin which was used as a light counterstain.

### 2.14. Statistical Analysis

Values are presented as the mean ± standard deviation (SD). All statistical analyses were performed using the Statistical Package for Social Science software, vers. 18 (SPSS, Chicago, IL, USA), and quantified data were analyzed using GraphPad Prism 7 (GraphPad Software Inc, San Diego, CA, USA). Differences between two groups were analyzed using Student’s *t*-test and were considered significant at *p* < 0.05.

## 3. Results

### 3.1. Penfluridol Treatment Alters Energy Metabolism in NSCLC Cells

**Figure 1:**
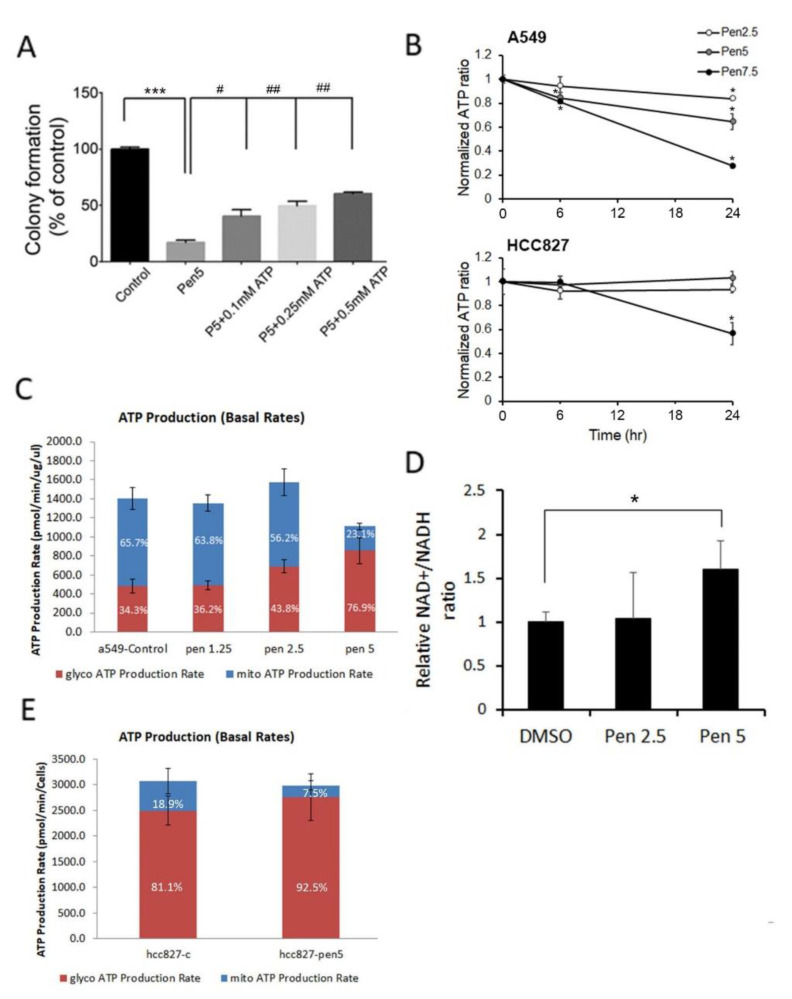
Effect of penfluridol on energy metabolism in human non-small cell lung cancer (NSCLC) cells. (**A**) A549 cells were treated with penfluridol (5 μM) combined with or without ATP (0.1, 0.25, or 0.5 mM) for 24 h; then, the antigrowth effects of penfluridol on cells were determined by counting the colonies formed. Values are presented as the mean ± SD from three independent experiments. *** *p* < 0.001 compared to the vehicle group. ^#^ *p* < 0.05, ^##^ *p* < 0.01, compared to the penfluridol-treated group. (**B**) Normalized ratio of ATP amount and cell viability in A549 and HCC827 cells treated with various concentrations of penfluridol (0, 2.5, 5, and 7.5 μM) for indicated time points. * *p* < 0.05 compared to the vehicle group. (**C**,**E**) Quantification of ATP production by a Seahorse XF real-time ATP rate assay following penfluridol treatment for 24 h in A549 (**C**) and HCC827 cells (**E**). Metabolic flux analysis showing quantification of mitochondrial ATP production and glycolytic ATP production. The percentages of mitochondrial ATP production and glycolytic ATP were shown on the bars. (**D**) The relative ratio changes of NAD+/NADH in A549 cells treated with penfluridol (2.5 and 5 µM) for 24 h. Data are shown as the mean ± SD from three independent experiments. * *p* < 0.05 compared to the vehicle group.

In our previous study, we demonstrated that penfluridol inhibits lung tumor growth via inducing ATP energy loss [23]. Herein, we observed that replenishment of ATP in penfluridol-treated A549 cells rescued inhibition of the colony-forming ability caused by penfluridol (Figure 1A) in a concentration-dependent manner, suggesting that the anticancer effect of penfluridol may be related to an energy shortage. To further determine the effect of penfluridol on ATP production in NSCLC cells, A549 and HCC827 cells were treated with various concentrations of penfluridol for 6 and 24 h, and the cell viability and total ATP amount were determined in parallel. We found that 24 h of treatment of both cells with penfluridol (5 or 7.5 µM) induced similar trends of inhibition of cell survival and ATP production (Figure S1A,B). After normalizing ATP levels to cell viability, the adjusted results also showed that ATP amounts dramatically decreased in both NSCLC cell lines with 24 h of treatment with penfluridol (5 or 7.5 µM) (Figure 1B). Interestingly, we found that the reduction in ATP in A549 cells was more sensitive to penfluridol treatment than it was in HCC827 cells. As we know, endogenous ATP is mainly generated from glycolysis and mitochondrial OXPHOS [27]. To further dissect the effect of penfluridol on energy metabolism, we applied a real-time ATP metabolism assay to compare changes in ATP production in mitochondrial OXPHOS and glycolysis. After treatment of A549 cells with various concentrations of penfluridol for 24 h, 5 µM penfluridol dominantly inhibited mitochondrial ATP production, but partially increased ATP generated by glycolysis (Figure 1C). NADH is an essential redox factor primarily responsible for ATP production by mitochondria [28]. We also observed that treatment of A549 cells with penfluridol induced downregulation of NADH and further caused an increase in the NAD+/NADH ratio (Figure 1D). These results suggested that penfluridol-mediated ATP deprivation mainly occurred through inhibition of mitochondrial OXPHOS, and glycolysis induction might be the source of compensatory energy acquisition in A549 cells. In HCC827 cells, although endogenous ATP production mainly depends on glycolysis (>80%), we also observed that ATP produced from mitochondria decreased and glycolysis yielded more ATP to compensate for the insufficient ATP in HCC 827 cells treated with 5 µM penfluridol (Figure 1E).

### 3.2. Penfluridol-Triggered Loss of Mitochondrial Function and Decrease in Mitochondrial Biogenesis via Downregulating PGC-1α in NSCLC Cells

**Figure 2:**
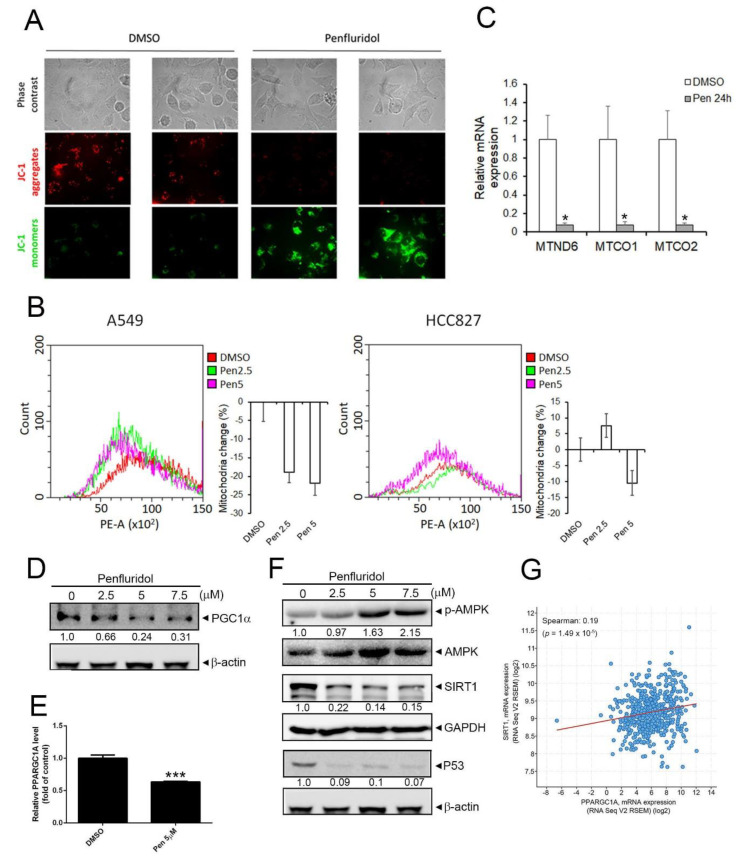
Penfluridol decreases mitochondrial activity and mass in human non−small cell lung cancer (NSCLC) cells via targeting peroxisome−proliferator−activated receptor co−activator−1α (PGC−1α). (**A**) Fluorescent JC−1 images of the mitochondrial membrane potential in A549 cells treated with vehicle or penfluridol (5 µM) for 24 h. The red−to−green fluorescence ratio indicates a decrease in functional mitochondria with the membrane potential. (**B**) For the flow cytometric analysis, cells were stained with MitoTracker Red H2XRos (which fluoresces upon oxidation in respiring mitochondria). Representative histograms (left panel) and bar graph *(n* = 3) (right panel) of the flow cytometric analysis between vehicle− and penfluridol−treated A549 and HCC827 cells are depicted. (**C**,**E**) A549 cells were treated with vehicle or penfluridol (5 µM) for 24 h to detect mRNA levels of MTND6, MTCO1, MTCO2 (**C**), and PPARGC1A (**E**) using an RT−qPCR. Quantitative results of indicated mRNA levels were adjusted to GAPDH mRNA levels. * *p* < 0.05, *** *p* < 0.001, compared to the vehicle group. (**D**,**F**) A549 cells were treated with vehicle or penfluridol at indicated concentrations for 24 h. Cells were harvested and then subjected to a Western blot analysis to detect PGC−1α (**D**), phosphorylated (p)−AMPK, sirtuin 1 (SIRT1), and p53 levels (**F**). GAPDH, β−actin, or AMP−activated kinase (AMPK) was used as an equal loading control. Western blots shown are representative results from 3 independent experiments. (**G**) Correlation analysis of the TCGA lung cancer database (TCGA, PanCancer Atlas) using the cBioPortal revealing a positive correlation between PPARGC1A and SIRT1.

Previous reports indicated that mitochondria use oxidizable substrates to produce an electrochemical proton gradient across mitochondrial membranes which is used to produce ATP, and the MMP is a key indicator of mitochondrial activity, because it reflects the process of electron transport and OXPHOS, the driving force behind ATP production [29]. Therefore, we next investigated the effect of penfluridol on the MMP and observed that penfluridol treatment caused a decrease in the MMP in A549 cells (Figure 2A). We further determined the effect of penfluridol on the mitochondrial mass using MitoTracker fluorescent dye. Treatment of A549 cells with 2.5 and 5 μM penfluridol for 24 h reduced the mitochondrial mass, but only 5 μM penfluridol treatment attenuated the mitochondrial mass in HCC827 cells (Figure 2B), suggesting that HCC827 cells were less responsive to penfluridol-mediated inhibition of mitochondrial biogenesis. In addition, mitochondrial genes coding for respiratory chain complexes, including MTND6, MTCO1, and MOTC2, were all significantly downregulated in A549 cells after 24 h of treatment with 5 μM penfluridol (Figure 2C). Taken together, these results indicated that penfluridol inhibited mitochondrial ATP production through suppressing mitochondrial activity and biogenesis. PGC-1α has been extensively described as a master regulator of mitochondrial biogenesis [10]. Western blot and RT-qPCR results revealed that PGC-1α protein and mRNA levels both decreased in penfluridol-treated A549 cells (Figure 2D,E). Both AMPK-mediated phosphorylation and SIRT1-mediated deacetylation of PGC-1α are two kinds of posttranslational modifications (PTMs) that increase PGC-1α activity [10]. Moreover, PGC-1α was reported to be transcriptionally regulated by wild-type (WT) p53 in lung cancer and neuroblastoma cells [30,31]. In penfluridol-treated A549 cells, we found that SIRT1 and p53 were significantly downregulated by penfluridol treatment, but phosphorylated (p)-AMPK was concentration-dependently upregulated by penfluridol (Figure 2F), suggesting that penfluridol may regulate PGC-1α in NSCLC cells via transcriptional regulation and PTMs. We analyzed 566 clinical NSCLC human samples that were retrieved from TCGA using the cBioportal platform and observed that PGC-1α (PPARGC1A) expression was significantly correlated with SIRT1 (SIRT1) (Figure 2G).

### 3.3. Inhibition of Glycolysis by 2DG Promotes the Growth Inhibitory Effect of Penfluridol in NSCLC Cells

**Figure 3:**
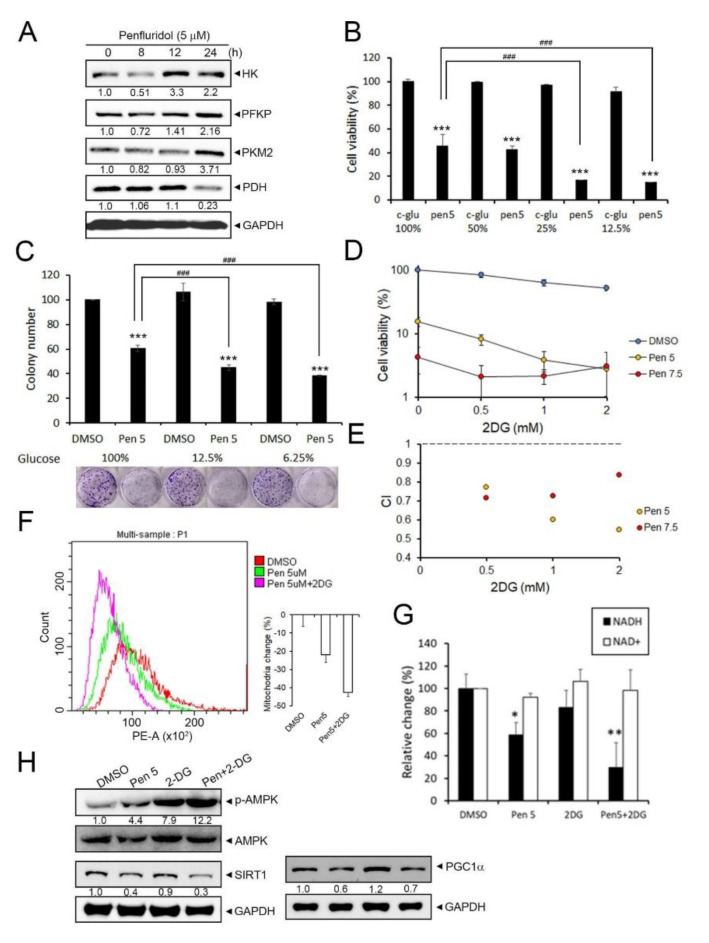
2−Deoxy−D−glucose (2DG) combined with penfluridol significantly reduces cell growth ability, which indicates a synergetic interaction between 2DG and penfluridol for human non−small cell lung cancer (NSCLC) cells. (**A**) A549 cells were treated with vehicle or penfluridol for the indicated time points and then subjected to a Western blot analysis to detect glycolysis−related markers. GAPDH was used as an equal loading control. Western blots shown are representative results from 3 independent experiments. (**B**,**C**) A549 cells were treated with vehicle or 5 µM penfluridol and cultured in complete medium containing indicated concentration of glucose (100%~6.25%), and the proliferative (**B**) and colony−forming (**C**) abilities were determined. Values are presented as the mean ± SD from three independent experiments. *** *p* < 0.001 compared to the vehicle group. ^###^ *p* < 0.001 compared to penfluridol−treated cells cultured in medium with 100% glucose. (**D**,**E**) A549 cells were treated with 5 or 7.5 μM of penfluridol with various concentrations of 2DG for 24 h. Then, the cell viability was determined by an MTS assay (**D**), and values of the combination index (CI) were calculated using the Compusyn software package (**E**), which were interpreted as follows: >1 antagonism, <1 synergism, and =1 additive. (**F**) For the flow cytometric analysis, cells were stained with MitoTracker Red H2XRos. A representative histogram (left panel) and bar graph (*n* = 3) (right panel) of the flow cytometric analysis among vehicle−, penfluridol (5 µM)−, and 2DG (1 mM)+penfluridol−treated A549 cells are depicted. (**G**) Changes in NAD+ and NADH in A549 cells treated with vehicle, penfluridol (5 µM), 2DG (1 mM), or penfluridol+2DG for 24 h. Data are shown as the mean ± SD from three independent experiments. * *p* < 0.05, ** *p* < 0.01 compared to the vehicle group. (**H**) A549 cells were treated with vehicle, penfluridol, 2DG, or penfluridol+2DG for 24 h and then subjected to a Western blot analysis to detect expression levels of phosphorylated (p) −AMP−activated kinase (AMPK), sirtuin 1 (SIRT1), and peroxisome−proliferator−activated receptor co−activator−1α (PGC−1α). GAPDH or AMPK was used as an equal loading control. Western blots shown are representative results from 3 independent experiments.

We further confirmed the effect of penfluridol on glycolysis by checking several key glycolytic enzymes and found that HK, PFKP, and PKM2 were upregulated by penfluridol treatment in A549 cells in a time-dependent manner, but PDH was downregulated (Figure 3A), suggesting that penfluridol actually triggered glycolysis in NSCLC cells. To determine whether the glucose concentration in the medium was a factor influencing the anticancer effect of penfluridol, we changed the concentration of glucose (100%~6.25%) and found that the inhibitory effect of penfluridol on proliferative (Figure 3B) and colony-forming (Figure 3C) abilities of A549 were all enhanced in cells cultured with low-glucose medium (25%, 12.5%, or 6.25% glucose) compared with cells cultured in medium with 100% glucose concentration. We next chemically induced a state of glucose deprivation using 2DG and found that combining penfluridol treatment with 2DG increased the penfluridol-induced cytotoxicity in A549 and HCC827 cells (Figure 3D and Figure S2A). Moreover, statistical analysis indicated that a synergy was present between the two drugs at 5 or 7.5 µM of penfluridol and all concentrations tested of 2DG (0.5~2 mM) in A549 cells, as the drug combination index (CI) values calculated with the software Compusyn were all <1 (Figure 3E). This synergistic effect of the combination of penfluridol (5 µM) and 2DG (0.5 or 1 mM) was also observed in HCC827 cells (Figure S2A,B). In addition, decreases in the mitochondrial mass (Figure 3F) and NADH levels (Figure 3G) caused by penfluridol were also enhanced when cells were cotreated with 2DG, suggesting that inhibition of glycolysis could enhance penfluridol-induced inhibition of mitochondrial biogenesis. AMPK is a known sensor of a decreased ATP/AMP ratio, and we found that penfluridol treatment partially induced AMPK phosphorylation, and cotreatment of 2DG dramatically increased AMPK phosphorylation in A549 cells (Figure 3H), suggesting that 2DG combined with penfluridol drastically enhanced ATP depletion. However, cotreatment of 2DG with penfluridol did not influence the penfluridol-mediated downregulation of SIRT1 or PGC-1α (Figure 3H).

### 3.4. The SIRT1/PGC-1α Axis Is Critical for Penfluridol-Regulated Mitochondrial Biogenesis and Cell Viability of NSCLC Cells

**Figure 4:**
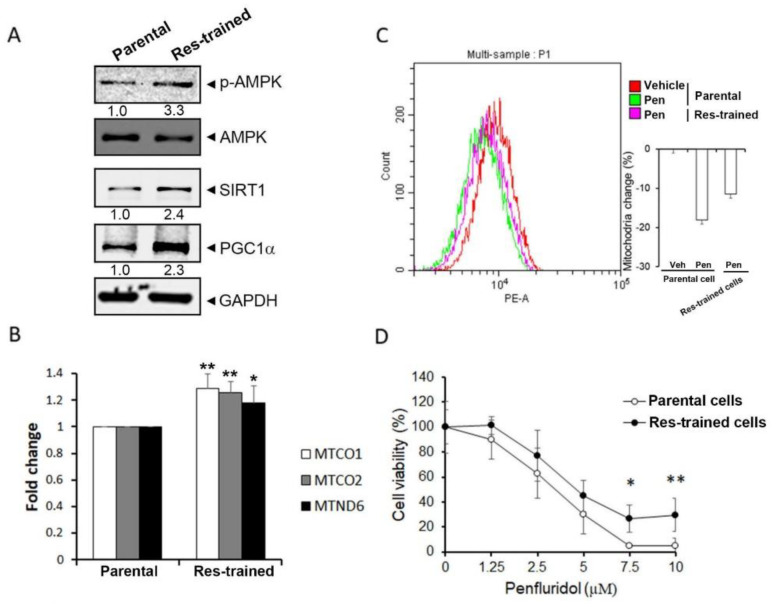
Upregulation of the sirtuin 1 (SIRT1)/peroxisome−proliferator−activated receptor co−activator−1α (PGC−1α) axis reverses penfluridol−mediated inhibition of cell proliferation and mitochondrial biogenesis. (**A**) A549 cells were treated with 50 µM resveratrol for 6 h per day for 3 days (resveratrol−trained cells), and a Western blot analysis was performed to detect levels of phosphorylated (p)−adenosine monophosphate−activated protein kinase (AMPK), SIRT1, and PGC−1α. GAPDH or AMPK was used as an equal loading control. Western blots shown are representative results from 3 independent experiments. (**B**) The mRNA levels of MTND6, MTCO1, and MTCO2 in resveratrol−trained and parental A549 cells were detected by using RT−qPCR. Quantitative results of indicated mRNA levels were adjusted to GAPDH mRNA levels. * *p* < 0.05, ** *p* < 0.01 compared to the parental cell group. (**C**) Resveratrol−trained and parental A549 cells were treated with penfluridol (5 µM) for 24 h to detect the mass of mitochondria by using flow cytometric analysis. A representative histogram (left panel) and bar graph (*n* = 3) of the flow cytometric analysis among vehicle− and penfluridol (5 µM)−treated parental cells and penfluridol−treated resveratrol−trained cells are depicted. (**D**) Resveratrol−trained and parental A549 cells were treated with various concentrations of penfluridol for 24 h. Then, the cell viability was determined by an MTS assay. * *p* < 0.05, ** *p* < 0.01 compared to the parental cell group. Res—resveratrol; Veh—vehicle.

Previous reports indicated that resveratrol can induce SIRT1 expression and was indicated as an SIRT1 activator in NSCLC cells including A549 cells [32]. Moreover, resveratrol was reported to improve mitochondrial function and protect against metabolic disease by activating SIRT1 and PGC-1α [33]. Furthermore, liver kinase B1 was demonstrated as a direct activator of SIRT1 elicited by resveratrol to trigger PGC-1α-mediated mitochondrial biogenesis [34]. Herein, A549 cells were pretreated with 50 µM resveratrol for 6 h per day for 3 days to establish cells that expressed higher levels of the SIRT1/PGC-1α axis. Actually, we observed that resveratrol-trained A549 cells showed higher protein levels of p-AMPK, SIRT1, and PGC-1α compared with the parental cells (Figure 4A). Moreover, the mitochondrial genes, MTCO1, MTCO2, and MTND6, were all significantly upregulated in resveratrol-trained cells (Figure 4B). Furthermore, the inhibitory effects of penfluridol on mitochondrial biogenesis (Figure 4C) and cell proliferation (Figure 4D) were significantly attenuated in resveratrol-trained A549 cells compared with parental cells, suggesting that the SIRT1/PGC-1α axis is a critical target of penfluridol for mediating suppression of mitochondrial biogenesis and cell growth in NSCLC cells.

### 3.5. Penfluridol in Combination with 2DG Dramatically Suppresses Tumor Progression in an A549 Orthotopic Graft Model

**Figure 5:**
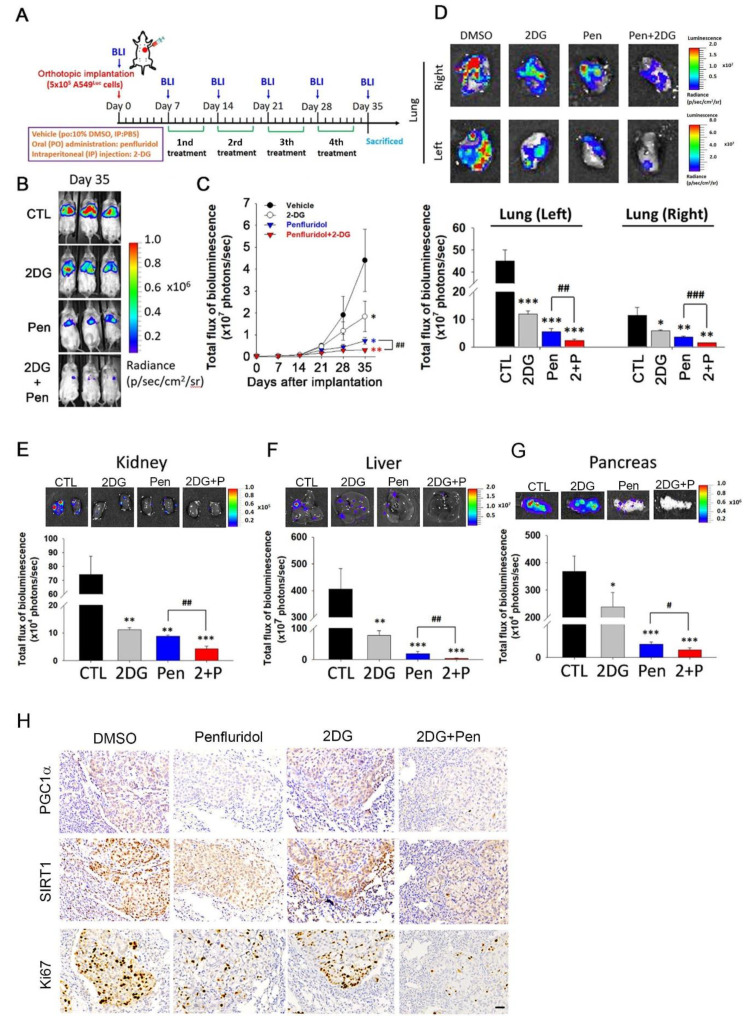
2−Deoxy−D−glucose (2DG) promotes the oncostatic effect of penfluridol in an A549 orthotopic graft model. (**A**) Timeline of the in vivo study design for investigating the antitumor activity of penfluridol, 2DG, and penfluridol+2DG on tumor progression. Male NOD/SCID mice were orthotopically injected with luciferase−tagged A549 cells. After 7 days, mice were treated with penfluridol (5 mg/kg, orally), 2DG (100 mg/kg, intraperitoneally), penfluridol+2DG, or the vehicle 5 days/week for 4 consecutive weeks. All mice were sacrificed at 4 weeks after drug treatment, and the luciferase activity was detected every week with a noninvasive imaging system (IVIS imaging system, Xenogen). (**B**,**C**) Bioluminescence imaging of orthotopic lung tumor growth at the end of the experiment (**B**). Quantitative analysis of Xenogen imaging signal intensity at the indicated time points (**C**). * *p* < 0.05; ** *p* < 0.01 compared to the vehicle control group; ^##^ *p* < 0.01 compared to the penfluridol-treated group. (**D**) Lungs were isolated and examined at the end of the experiment. Upper panel: Cancer metastasis from the left lung to the right lung was imaged with bioluminescence at the end of the experiment. Lower panel: Signal intensities from primary tumors (left lung) and metastatic tumors (right lung) were captured with bioluminescence at the end of the experiment, with the mean signal for each group indicated. * *p* < 0.05, ** *p* < 0.01, *** *p* < 0.001 compared to the vehicle control group. ^##^ *p* < 0.01, ^###^ *p* < 0.001, compared to the penfluridol-only-treated group. (**E**–**G**) Representative ex vivo bioluminescence imaging of distal metastatic sites. Signal intensities from distal metastatic organs including the kidneys (**E**), liver (**F**), and pancreas (**G**) were captured with bioluminescence, with the mean signal for each group indicated. * *p* < 0.05, ** *p* < 0.01, *** *p* < 0.001 compared to the vehicle control group. ^#^ *p* < 0.01, ^##^ *p* < 0.001, compared to the penfluridol−only−treated group. (**H**) A549 xenografts treated with indicated drugs were isolated to detect expressions of Ki67, sirtuin 1 (SIRT1), and peroxisome−proliferator−activated receptor co-activator-1α (PGC−1α) by IHC staining. Original magnification, 400×. Scale bar, 30 μm.

To further explore the feasibility of the combination regimen in vivo, we established an orthotopic lung-tumor-bearing model by transplanting luciferase-tagged A549 cells into NOD-SCID mice and allowed them to become established for 7 days before initiating treatment. The experimental group of mice was treated with 5 mg/kg penfluridol by oral gavage, 100 mg 2DG/kg BW by IP administration, or a combination of these two drugs 5 days/week, whereas mice in the control group only received the vehicle. A schematic timeline of this experimental design and setup is shown in Figure 5A. Effects of the administration of penfluridol, 2DG, and both drugs on tumor growth and metastasis were monitored by bioluminescence imaging. In vivo photon emission detection revealed that penfluridol and 2DG treatments alone both inhibited tumor growth, while penfluridol+2DG combination treatment decreased tumor growth more significantly (Figure 5B,C). Comparable to in vivo data, ex vivo imaging of the lungs from sacrificed mice revealed lower photon intensities in penfluridol- or 2DG-treated mice compared with vehicle-treated mice, and the combination regimen of both drugs showed higher inhibitory potential than either drug in both orthotopic tumors (in the left lung) and metastatic tumors (in the right lung) (Figure 5D). In addition to lung tissues, distant metastases of cells to other organs such as the kidneys (Figure 5E), liver (Figure 5F), and pancreas (Figure 5G) were all significantly suppressed by penfluridol or 2DG treatment, and the penfluridol+2DG combination still exhibited the strongest inhibitory effect. To further examine the effects of penfluridol and 2DG treatment on proliferation and the related mechanisms, an IHC analysis was used to determine the anticancer effects of penfluridol, 2DG, and cotreatment of both drugs. We observed that lower Ki67 was induced by penfluridol or 2DG treatment, and especially in penfluridol+2DG cotreatment (Figure 5H). In contrast to Ki67, lower PGC-1α and SIRT1 expressions were only observed in the penfluridol-treated group, and cotreatment with 2DG+penfluridol did not enhance the inhibitory effect of penfluridol on the SIRT1/PGC-1α axis (Figure 5H).

## 4. Discussion

This study aimed to investigate novel mechanisms underlying the anticancer efficacy of penfluridol in NSCLC. Our previous results showed that penfluridol (7.5 or 10 μM) suppressed cell proliferation of NSCLC cells via inducing endoplasmic reticulum (ER) stress-mediated autophagosome accumulation to deplete ATP energy [23]. Herein, we further identified that penfluridol mediated downregulation of ATP through inhibiting mitochondrial OXPHOS via inducing loss of the MMP and decreasing mitochondrial biogenesis. We found that the SIRT1/PGC-1α axis is a critical target in penfluridol-regulated mitochondrial biogenesis. Meanwhile, penfluridol also induced glycolysis to compensate for the loss of ATP caused by suppression of mitochondrial OXPHOS. Therefore, we found that in combination of the glycolysis inhibitor, 2DG, and penfluridol (5 or 7.5 µM) further enhanced the anticancer potential of this repurposed drug in NSCLC cells.

In this study, we used two NSCLC cell lines which, respectively, harbor mutant KRAS (A549) and mutant EGFR (HCC827). Interestingly, we observed that A549 cells relied more on mitochondrial OXPHOS to produce ATP than did HCC827 cells and were more sensitive to ATP loss caused by penfluridol. These results suggest that targeting mitochondrial metabolism by penfluridol is more suitable for treating mutant KRAS-expressing NSCLC cells than mutant EGFR-expressing cells. Actually, mitochondrial metabolism was reported to be essential for KRAS-induced cell proliferation and tumorigenesis [7]. Recently, a transcription factor, sterol regulatory element-binding protein 1 (SREBP1), was reported to affect mitochondrial OXPHOS to modulate proliferative ability, but did not affect the biogenesis of mitochondria or glycolysis in mutant KRAS-expressing NSCLC cell lines such as A549 cells [35]. Whether SREBP1 plays a role in penfluridol-induced inhibition of mitochondria OXPHOS in mutant KRAS-expressing NSCLC cells needs to be investigated in the future.

PGC-1α is a critical controller of energy metabolism, which is achieved by acting on both mitochondrial biogenesis and OXPHOS in different cancer types including NSCLC [36]. Ectopic expression of PGC-1α was observed in several cancer types [10], and the increased expression of PGC-1α was reported to be associated with low survival rates of NSCLC patients [37]. Our study showed that penfluridol can suppress mitochondrial biogenesis via decreasing mRNA and protein levels of PGC-1α in A549 cells. PGC-1α was reported to be regulated by several oncogenes and signaling pathways through transcriptional regulation or PTMs. For example, SIRT1 was shown to increase PGC-1α expression and activity through promoting transcription [38] and deacetylation [10], suggesting that SIRT1 can regulate PGC-1α through both transcriptional and posttranslational regulation. Herein, we showed that penfluridol suppressed SIRT1 expression in A549 cells in a concentration-dependent manner, and upregulation of the SIRT1/PGC-1α axis significantly reversed the inhibitory effect of penfluridol on mitochondrial biogenesis and cell proliferation. These results suggested that the SIRT1/PGC-1α axis may facilitate NSCLC growth by promoting mitochondrial biogenesis, and penfluridol is a potential inhibitor for blocking this pathway. Moreover, previous studies indicated that chemotherapy of cancers induced an SIRT1/PGC-1α axis-dependent increase in OXPHOS that promoted tumor survival against chemotherapeutic drugs [12,36], suggesting that penfluridol exhibits the potential to increase the chemosensitivity of NSCLC cells, and this issue should be further investigated. In addition to SIRT1, our results showed that another PGC-1α regulator, p53, was also inhibited by penfluridol in A549 cells. PGC-1α expression was reported to be influenced by WT p53. Taguchi et al. evaluated mRNA levels of PGC-1α in 28 NSCLC cell lines with different p53 mutational statuses and observed that mRNA levels of PGC-1α were higher in p53 WT cells compared with cell lines harboring p53 loss or missense mutations [30], implying that PGC-1α might be a potential target of WT p53 in NSCLC. In fact, the direct transcription regulation of WT p53 by PGC-1α was observed in neuroblastoma cells [31]. A549 cells are also an NSCLC cell line harboring WT p53; therefore, penfluridol-induced transcriptional inhibition of PGC-1α may also occur through p53 inhibition.

Our results showed that the inhibitory effect of penfluridol on mitochondrial OXPHOS led to compensatory glycolysis in NSCLC cells. A previous study indicated that a cell death and survival signaling factor, receptor-interacting protein 1 (RIP1), regulated mitochondrial OXPHOS, and aerobic glycolysis. Chen et al. indicated that knockdown of RIP1 suppressed PGC-1α expression and mitochondrial OXPHOS, resulting in accelerated glycolysis in A549 cells. Moreover, the viability of A549 cells with RIP1-knockdown was more sensitive to glycolysis inhibition with 2DG [39]. Our results also showed that cotreatment with 2DG enhanced penfluridol-mediated inhibition of cell viability. Whether RIP1 plays a role in penfluridol-regulated PGC-1α expression, energy metabolism, and viability suppression in NSCLC cells needs to be further investigated.

It is well known that AMPK is a key cellular sensor of reduced energy supply and as such is activated by increases in the intracellular ratio of AMP/ATP. Previous studies indicated that upregulation of glycolysis in response to inhibition of mitochondrial ATP synthesis was dependent on AMPK activity in NSCLC cells including A549 cells [40]. Moreover, 6-phosphofructo-2-kinase/fructose-2,6-biphosphatase 3 (PFKFB3) was reported to be a critical downstream target of activated AMPK to induce glycolysis in cancer cells [41], including A549 cells [42]. Our results showed that AMPK activation (phosphorylation) was induced by penfluridol in A549 cells in a concentration-dependent manner, suggesting that the AMPK/PFKFB3 axis may be involved in penfluridol-triggered glycolysis in NSCLC cells. Therefore, direct inhibition of AMPK may be another way to enhance the anticancer activity of penfluridol in NSCLC cells.

## 5. Conclusions

**Figure 6:**
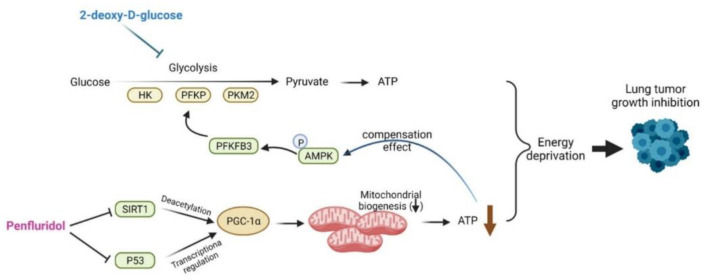
A working model showing the molecular mechanisms underlying the ability of penfluridol to modulate energy metabolism of human non-small cell lung cancer (NSCLC) cells. ATP deprivation induced by penfluridol was attributed to inhibition of mitochondrial oxidative phosphorylation (OXPHOS) via inducing loss of the mitochondrial membrane potential (MMP) and a decrease in mitochondrial biogenesis. The sirtuin 1 (SIRT1)/peroxisome-proliferator-activated receptor co-activator-1α (PGC-1α) axis is critical for penfluridol-regulated mitochondrial biogenesis. Meanwhile, penfluridol also induced adenosine monophosphate-activated protein kinase (AMPK)-mediated glycolysis to compensate for the loss of ATP caused by mitochondrial OXPHOS suppression. A combination of the glycolysis inhibitor, 2DG, and penfluridol further enhanced the anticancer potential of this repurposed drug in NSCLC cells.

In summary, we report that penfluridol, a drug for treating schizophrenia in the clinic, can suppress mitochondrial OXPHOS through targeting the SIRT1/PGC-1α axis to reduce ATP production, especially in mutant KRAS-expressing NSCLC cells. In response to penfluridol-induced ATP depletion, NSCLC cells may turn to AMPK/PFKFB3-mediated compensatory glycolysis. Herein, we first report that blockage of glycolysis by 2DG can enhance the inhibitory effect on cancer cell growth by penfluridol in NSCLC cells. The proposed signal transduction pathways by which penfluridol mediates energy metabolism of NSCLC cells are schematically illustrated in Figure 6.

## Data Availability

All data used during the current study are available from the corresponding author upon reasonable request.

## Supplementary Materials

The following supporting information can be downloaded at: https://www.mdpi.com/article/10.3390/cancers14112750/s1, Figure S1: Effect of penfluridol on cell survival and ATP production in non-small cell lung cancer (NSCLC) cells, Figure S2: 2-Deoxy-D-glucose (2DG) potentiates the growth inhibitory effect of penfluridol in HCC827 cells.
