# Supplementary material for: Synergistic Tumor Inhibition via Energy Elimination by Repurposing Penfluridol and 2-Deoxy-D-Glucose in Lung Cancer
Source: Cancers (Basel). 2022 Jun 1;14(11):2750. doi: 10.3390/cancers14112750 (PMC9179427; doi:10.3390/cancers14112750)
Supplement: Supplementary file 1 [file cancers-14-02750-s001.zip › cancers-1697621-supplementary.pdf]

cancers

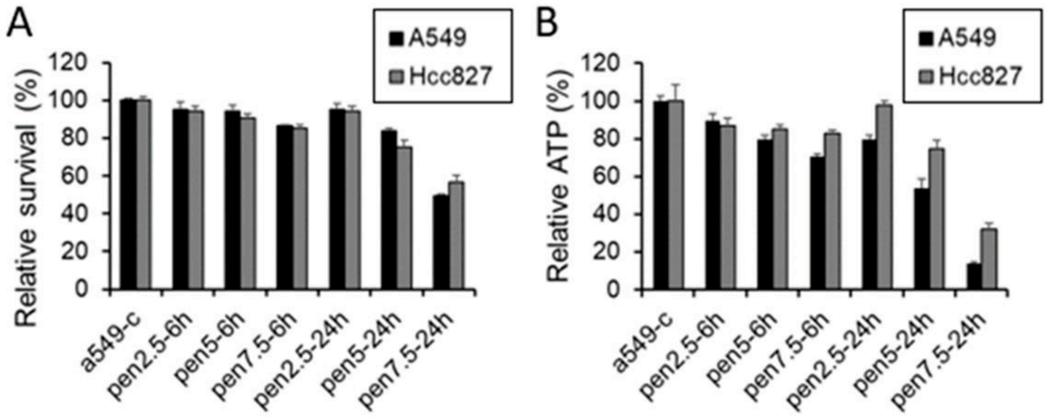

Figure S1. Effect of penfluridol on cell viability and ATP production in human non-small-cell lung cancer (NSCLC) cells. A549 and HCC827 cells were treated with indicated concentrations of penfluridol for 6 or 24 h, and then subjected to an MTS assay to determine the cell viability (A) or a luminescent ATP detection assay to detect intracellular ATP.

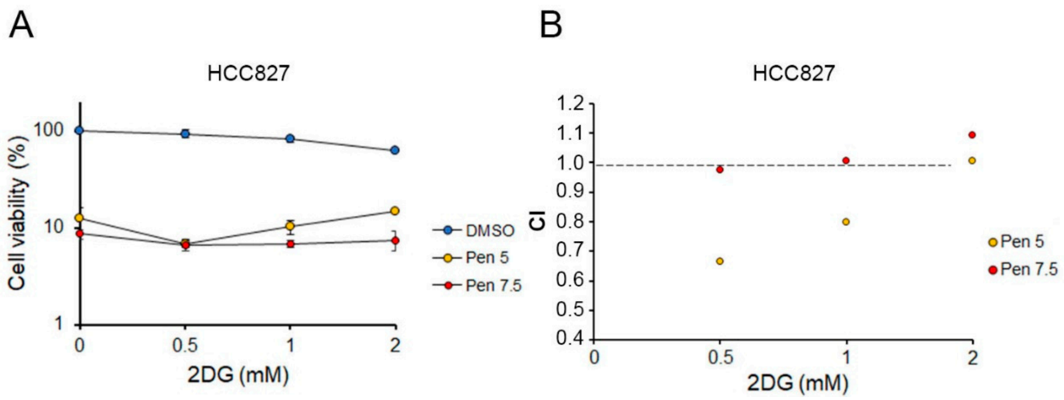

Figure S2. Penfluridol combined with 2-deoxy-D-glucose (2DG) enhance growth inhibition in human non-small-cell lung cancer (NSCLC) cells. HCC827 cells were treated with 5 or 7.5 µM of penfluridol with various concentrations of 2DG for 24 h. The cell viability was determined by an MTS assay (A), and values for the combination index (CI) were calculated using the Calcusyn software package (B), which were interpreted as follows: >1 antagonism, <1 synergism, and =1 additive.
